# Obesity, waist circumference, and appendicular muscle mass ratio in relation to blood pressure among the community‐dwelling elderly population in Taiwan

**DOI:** 10.1111/jch.14429

**Published:** 2022-01-28

**Authors:** Chun‐Yung Chang, Chih‐Chun Kuo, Ming‐Hsun Lin, Der‐Min Wu, Chieh‐Hua Lu, Nain‐Feng Chu

**Affiliations:** ^1^ Department of Internal Medicine Kaohsiung Armed Forces General Hospital Kaohsiung Taiwan ROC; ^2^ Division of Endocrinology and Metabolism, Department of Internal Medicine, Tri‐Service General Hospital National Defense Medical Center Taipei Taiwan; ^3^ Department of Internal Medicine TaoYuan Armed Forces General Hospital TaoYuan Taiwan ROC; ^4^ School of Public Health National Defense Medical Center Taipei Taiwan ROC

**Keywords:** appendicular muscle mass ratio, blood pressure, elderly, obesity, waist circumference

## Abstract

Hypertension is known to be related to obesity and both are the major factors for cardiovascular diseases. The relationship between body composition and blood pressure (BP) are discussed recently. Our study aims to evaluate the association between waist circumference (WC) and appendicular muscle mass (AMM) in relation to BP among the community‐dwelling elderly population.

Total 3739 patients (1600 males and 2139 females) were recruited in a series of community‐based surveys that were conducted among the elderly population in Taiwan from 2017 to 2019. We collected data on anthropometric characteristics, handgrip strength, and BP using standard methods. AMM was calculated with an equation. History of chronic disease and lifestyle profiles were collected using questionnaires.

The group with high AMM to body weight ratio (AMMW) showed lower systolic BP (SBP) (136.8 ± 19.1 to 140.6 ± 17.0 for males; 137.8 ± 18.3 to 142.7 ± 17.5 for females, both *P* < .001). Among central obese persons those with higher AMMW ratio had lower SBP. In the final model, AMMW in percentage is negatively associated to SBP (β = −0.641 in male, −0.780 in female, both *P* < .01). In other words, every 10% increase in AMMW is associated with decrease of SBP 6.41 mmHg in male and 7.80 mmHg in female.

Obesity and central obesity were positively associated with BP. The AMMW ratio was negatively associated with HTN and with a protective effect on BP even among the central obese. Health promotion programs to increase physical training may prevent hypertension among the elderly in Taiwan.

## 1 INTRODUCTION

Both hypertension and obesity are important risk factors for cardiovascular disease.^1^, ^2^ The relationship between hypertension and obesity had been established^3^ and previous studies also demonstrated the positive association of body mass index (BMI) to both systolic and diastolic blood pressure (BP) levels.^4^, ^5^

Despite general obesity, central obesity which is defined by waist circumference (WC) also plays an important role in cardiometabolic diseases.^6^ Studies have shown a positive association of gain of WC with increased BP.^7^, ^8^ The relationship between different measurements of obesity and hypertension reported that even the WC predicts the risk of hypertension better than BMI.^9^

Sarcopenia was defined as age‐related loss of muscle.^10^ Both an increase in adipose tissue and a decrease in muscle mass are key risk factors of cardiometabolic disease in the elderly.^11^ Recently, the concern for the relationship between sarcopenic obesity and BP has risen. Although earlier meta‐analysis and systemic review demonstrated sarcopenia is associated with hypertension,^12^ the direct relationship between lean body mass and BP remains inconsistent since several studies had demonstrated a positive association of lean body mass to BP.^13^, ^14^, ^15^ On the other hand, few studies in Asian population use lean body mass to total body mass ratio as indicator and revealed an inverse relationship with BP.^16^, ^17^ In our study, we also included AMM to total body mass ratio and try to evaluate its relationship to BP.

The objective of this study was to evaluate the association of obesity, central obesity, AMM, and BP among the community‐dwelling elderly population in Taiwan. Therefore, by understanding the relationship between body composition and BP, our strategy toward physical training health promotion among the elderly can be improved in the future.

## 2 MATERIALS AND METHODS

### 2.1 Study population

A Series of community‐based surveys were conducted among elderly individuals in Chiayi County in Taiwan from 2017 to 2019. We invited those who were greater than and aged to 65 years and who have lived in Chiayi County for more than one year. The inclusion criteria were individuals aged 65–85 years and those free from infectious diseases or acute disorders in the past three weeks.

### 2.2 Questionnaire

General demographic data including gender, age, residency, education level, occupation, and the need for a caregiver were collected using a standard questionnaire. Lifestyle patterns including dietary habits, cigarette smoking, alcohol intake, and daily activity were also collected. History of chronic diseases including diabetes, hypertension, cardiovascular disease, chronic kidney disease, any type of cancer, and cerebrovascular disease as well as medication history was recorded by a research technician.

### 2.3 Anthropometric measurement

The anthropometric characteristics including body weight (BW), body height, and WC were determined using standard methods. The height was measured in meters using a digital stadiometer that recorded to the nearest 0.5 cm. BW was measured to an accuracy of 0.1 kg by a standard beam balance scale. During the above measurement, the persons were barefoot and they wore only light indoor clothing. The WC was measured using standard methods suggested by the World Health Organization. The WC was measured to the nearest 0.1 cm at the midpoint between the margin of the last rib and the iliac crest of the ilium. BMI was calculated by dividing BW (kg) by the square of the height (m^2^).

### 2.4 Appendicular muscle mass (AMM) measurement

The equation to calculate AMM included handgrip strength (GS), BW, sex, and height:

$$\begin{array}{ccc} \mathrm{AMM} & = & -9.833+0.397\times \mathrm{weight}\left(\mathrm{kg}\right)+4.433\times \mathrm{sex}\\ & & +\;0.121\times \mathrm{height}\left(\mathrm{cm}\right)+0.061\times \text{hand}\;\text{GS}\left(\mathrm{kg}\right). \end{array}$$

This equation best predicts AMM measured by dual‐energy X‐ray absorptiometry (adjusted R^2^ = 0.914, standard error of the estimate = 2.062, *P* < .001).^18^ As BMI, WHR, and AMM, may confound with each other. We found the AMM divided by the total body weight ratio as a surrogate marker of the percentage of body lean muscle (as AMMW) may demonstrate different body composition meaning. We also calculated the AMMW ratio (Consequently, the study population was divided into two subgroups based on the median of AMMW (0.647 for males, 0.570 for females). The group with a higher AMMW ratio was defined as AMMW (+) and the group with a lower AMMW ratio was defined as AMMW (−).

### 2.5 Blood pressure measurement

Blood pressures were measured after resting 5‐10 minutes in a seated position. The right arm was asked to be positioned into cuffs of appropriate sizes at the same height as the heart. Two measurements were recorded; the mean value of the two recordings was used for data analysis. The pulse pressure (PP) was calculated by systolic blood pressure (SBP) minus diastolic blood pressure (DBP). Mean arterial pressure (MAP) was calculated by one third of SPP plus two thirds of DBP.

### 2.6 Grip strength measurement

The GS was measured using digital dynamometers (TKK5101). All persons were in a seated position with fully extended elbows. After 2‐3 minutes of rest, we measured the GS for right or left hands two times. Two values for the GS were recorded, and the mean value of the two recordings was used for analysis.^19^

### 2.7 Approval of the IRB

All participants provided written informed consent and agreed to have their general demographic data, questionnaire, and anthropometric data taken for this study. The institutional review board of the Tri‐service General Hospital (Number: TSGHIRB‐1‐108‐05‐073) approved this study.

### 2.8 Statistical methods

We used SPSS v. 22 to conduct all statistical analyses. Continuous variables, such as anthropometric measures, blood pressure, and grip strength were presented as sample mean and SD. The Mann‐Whitney U test was used to compare the differences between the groups. The Kruskal‐Wallis H test was used to compare > 3 groups. The Pearson's correlation coefficient was applied for correlation of each variable. The categorical variables were described as numbers and percentages. The chi‐square test was performed to compare the differences among ≥ 2 groups. Multivariant regression analyses and logistic regression analyses were used for further statistical inference and a two‐tailed *P*‐value < .05 was considered statistically significant.

## 3 RESULTS

Table 1 demonstrates the general characteristics of anthropometric measures, BP, chronic disease status, and adverse lifestyle behaviors according to AMMW subgroups among the study male and female population, respectively. In this study, there was about 87.3% (603/691) male and 87.5 % (832/951) female with hypertension that took anti‐hypertension medications regularly. The study population was divided into two subgroups based on the median of AMMW with gender specification. The persons with higher AMMW showed lower SBP (136.8 ± 19.1 to 140.6 ± 17.0, *P* < .001 for male; 137.8 ± 18.3 to 142.7 ± 17.5, *P* < .001 for female); lower DBP (80.6 ± 10.9 to 82.8 ± 11.1, *P* < .001 for male; 77.4 ± 10.3 to 80.0 ± 10.0, *P* < .001 for female); lower MAP (99.3 ± 12.3 to 102.1 ± 11.5, *P* < .001 for male; 97.5 ± 11.4 to 100.9 ± 11.0, *P* < .001 for female), and lower prevalence of hypertension (35.6% to 50.7% in male and 36.6% to 52.3% in female, both *P* < .001).

**TABLE 1:** Characteristics of anthropometric measures, grip strength, blood pressure, chronic disease, and adverse behaviors among community‐based population (no. = 3739)

	AMMW(‐)	AMMW(+)	
Variables	Mean±SD	Mean±SD	*P*
Male (no. = 1600)			
Age (years)^a^	72.9±5.9	73.6±6.2	.038
Body height (cm)	163.0 6.1	161.7 6.0	<.001
Body weight (kg)	73.0 7.9	58.5 6.1	<.001
BMI (kg/m^2^)^a^	27.5±2.6	22.4±19	<.001
Body waist (cm)^a^	94.2±7.2	82.5±6.7	<.001
Grip strength (kg)	33.1±7.4	32.5±6.9	.123
AMM (kg)	45.3±3.8	39.4±3.2	<.001
AMMW	0.622±0.018	0.675±0.024	<.001
SBP (mmHg)	140.6±17.0	136.8±19.1	<.001
DBP (mmHg)	82.8±11.1	80.6±10.9	<.001
PP (mmHg)	57.9±14.3	56.2±14.9	.023
MAP (mmHg)	102.1±11.5	99.3±12.3	<.001
CVD (no.,%)^b^	140 17.5	112 14.0	.055
CVA (no.,%)^b^	38 4.8	24 3.0	.070
Hypertension (no.,%)^b^	406 50.7	285 35.6	<.001
DM (no.,%)^b^	201 25.1	133 16.6	<.001
CKD (no.,%)^b^	35 4.4	30 3.8	.527
Smoking (no.,%)^b^	94 11.8	119 14.9	.066
Alcohol drinking (no.,%)^b^	146 18.3	131 16.4	.322
Female (no. = 2139)			
Age (years)^a^	72.5±5.9	72.9±6.1	.118
Body height (cm)	150.5 5.6	151.3 5.8	.001
Body weight (kg)	63.5 7.8	50.8 6.0	<.001
BMI (kg/m^2^)^a^	28.0±2.9	22.1±1.9	<.001
Body waist^a^	88.8±7.9	77.5±6.9	<.001
Grip strength (kg)	21.4±4.8	21.8±4.8	.098
AMM (kg)	34.9±3.6	30.0±3.1	<.001
AMMW	0.551±0.014	0.591±0.017	<.001
SBP (mmHg)	142.7±17.5	137.8±18.3	<.001
DBP (mmHg)	80.0±10.0	77.4±10.3	<.001
PP (mmHg)	62.7±14.7	60.4±15.2	.001
MAP (mmHg)	100.9±11.0	97.5±11.4	<.001
CVD (no.,%)^b^	172 16.1	150 14.0	.180
CVA (no.,%)^b^	20 1.9	16 1.5	.500
Hypertension(no.,%)^b^	559 52.3	392 36.6	<.001
DM (no.,%)^b^	239 22.4	184 17.2	.003
CKD (no.,%)^b^	30 2.8	22 2.1	.260
Smoking (no.,%)^b^	8 0.7	11 1.0	.491
Alcohol drinking (no.,%)^b^	27 2.5	20 1.9	.300

Abbreviations: AMM, appendicular muscle mass; AMMW, appendicular muscle mass divided by body weight; BMI, body mass index; CKD, chronic kidney disease.; CVA, cerebrovascular disease; CVD, cardiovascular disease; DBP, diastolic blood pressure; DM, diabetes mellitus; MAP, mean arterial pressure; PP, pulse pressure; SBP, systolic blood pressure.

^a^ t test was compared with AMMW(−) and AMMW(+) among age, obesity characteristics, and lipids profiles; TG using log transformation to test.

^b^ chi‐square test was compared with AMMW(−) and AMMW(+) among chronic diseases and adverse behaviors.

Table 2 shows the correlation between anthropometric measures, AMM, AMMW, and different types of BP measurements among study population with gender specifications. For example, the BMI was positively correlated to both SBP (r = 0.166 in male, r = 0.176 in female, both *P* < .001) and DBP (r = 0.149 in male, r = 0.172 in female, both *P* < .001). The WC showed positive correlation to SBP (r = 0.158 in male, r = 0.153 in female, both *P* < .001) and DBP (r = 0.116 in male, r = 0.123 in female, both *P* < .001). The AMM was positively correlated to both SBP (r = 0.126 in male, r = 0.124 in female, both *P* < .001) and DBP (r = 0.213 in male, r = 0.181 in female, both *P* < .001). The AMMW ratio was negatively correlated to SBP (r = −0.168 in male, r = −0.167 in female, both *P* < .001) and DBP (r = −0.140 in male, r = −0.150 in female, both *P* < .001).

**TABLE 2:** Correlation between blood pressure and anthropometric measures, AMM, and AMM/W among community‐based population with gender specifications

	SBP (mmHg)	DBP (mmHg)	PP (mmHg)	MAP (mmHg)
	coefficient	coefficient	coefficient	coefficient
Male (no. = 1600)				
Age (years)	0.142***	−0.143***	0.284***	−0.017
BMI (kg/m^2^)	0.166***	0.149***	0.093***	0.175***
Body waist (cm)	0.158***	0.116***	0.109***	0.151***
Grip strength (kg)	−0.003	0.195***	−0.151***	0.118***
AMM (kg)	0.126***	0.213***	−0.005	0.194***
AMMW	−0.168***	−0.140***	−0.103***	−0.170***
Female (no. = 2139)				
Age (years)	0.188***	−0.089***	0.286***	0.046*
BMI (kg/m^2^)	0.176***	0.172***	0.094***	0.197***
Body waist (cm)	0.153***	0.123***	0.100***	0.155***
Grip strength (kg)	0.022	0.094***	−0.038	0.069***
AMM (kg)	0.124***	0.181***	0.026	0.175***
AMMW	−0.167***	−0.150***	−0.098***	−0.179***

Abbreviations: AMM, appendicular muscle mass; AMMW, appendicular muscle mass divided by body weight;BMI, body mass index; DBP, diastolic blood pressure; MAP, mean arterial pressure.; PP, pulse pressure; SBP, systolic blood pressure.

***P < .001, **P < .01, *P < .05.

Table 3 summarizes the distributions of BP among different WC and AMMW subgroups with gender specifications. Persons were further divided based on their WC (as central obesity, OB+: WC ≥ 90 cm for male, WC ≥ 80 cm for female; non‐central obesity, OB‐: WC < 90 cm for male, < 80 cm for female). AMMW was also divided into two subgroups according to the median of AMMW (0.647 for male and 0.570 for female). Then, the study persons were divided into four subgroups based on different AMMW and WC statuses. The subgroup with both central obesity and lower AMMW (OB(+), AMMW (−)) had the highest SBP (141.1 ± 17.1 for male,143.8 ± 18.2 for female), DBP (83.0 ± 11.4 for male, 80.4 ± 10.1 for female), and MAP (102.3 ± 11.8 for male, 101.5 ± 11.3 for female).

**TABLE 3:** Distributions (Mean±SD) of blood pressure among study population by specific anthropometrics, AMMW, and gender groups

	Obesity and AMMW Status					
	OB(−), AMMW(+) Group 1	OB(−), AMMW(−) Group 2	OB(+), AMMW(+) Group 3	OB(+), AMMW(−) Group 4	*P*‐value^a^	Post hoc
Male(no. = 1600)	(no. = 702)	(no. = 201)	(no. = 98)	(no. = 599)		
SBP(mmHg)	136.5±19.1	139.3±16.6	138.9±18.7	141.1±17.1	<.001	1 < 4^a^
DBP(mmHg)	80.6±10.9	82.2±10.3	80.9±11.3	83.0±11.4	.001	1 < 4^a^
PP(mmHg)	55.9±15.0	57.1±14.2	58.0±14.3	58.1±14.4	.353	
MAP(mmHg)	99.2±12.3	101.2±10.8	100.2±12.5	102.3±11.8	<.001	1 < 4^a^
Female(no. = 2139)	(no. = 1022)	(no. = 617)	(no. = 48)	(no. = 452)		
SBP(mmHg)	137.8±18.1	141.9±16.9	138.1±20.8	143.8±18.2	<.001	1 < 2,4^a^
DBP(mmHg)	77.4±10.2	79.8±10.0	76.6±12.7	80.4±10.1	<.001	1 < 2,4^a^
PP(mmHg)	60.4±15.2	62.2±14.6	61.4±15.9	63.4±15.0	.058	
MAP(mmHg)	97.5±11.3	100.5±10.7	97.1±14.0	101.5±11.3	<.001	1 < 2,4^a^

Abbreviations: DBP, Diastolic blood pressure; MAP, Mean arterial pressure.; PP, Pulse pressure;SBP, Systolic blood pressure.

OB(−), body waist < 90 cm for male or body waist < 80 cm for female; OB(+), body waist > = 90 cm for male or body waist > = 80 cm for female; AMMW(+), AMMW > = median for male and female; AMMW(−), AMMW < median for male and female, the median value of AMMW is 0.647 for male and 0.570 for female.

^a^ Post hoc test for paired comparison between groups, for example, 1 < 4 to demonstrate that the blood pressure in Group 4 [OB(+), AMMW(−)] was higher than the Group 1 [OB(−), AMMW(+)] with statistical significance.

Table 4 shows the association between AMMW on different BP measurements after adjusting for potential confounds with gender specification. In this study, we could not obtain the information on the type of anti‐hypertension medications. Anti‐hypertensive medicine may be potential cofounder and we only controlled this as with and without anti‐hypertensive medicine in the model. In the final model, after adjusting for age, body waist, disease status, and anti‐hypertensive medications, the AMMW was negatively associated with blood pressure. For example, every increase of 10% of AMMW is associated with decrease of 6.4 mmHg SBP and decrease of 3.1 mmHg DBP in male. The regression coefficients all are with statistical significance, such as, for SBP (β = −0.641 in male, *P* < .01; β = −0.780 in female, *P* < .001), DBP (β = −0.312 in male, *P* < .05; β = −0.438 in female, *P* = .001), and MAP (β = −0.422, *P* < .01 in male; β = −0.552, *P* < .001 in female).

**TABLE 4:** Multivariate regression analyses of AMMW (for every 1% change) on different blood pressure dependent variables with gender specifications

Dependent	Model I^a^			Model II^b^		
variables	β	se β	*P*‐value	Β	se β	*P*‐value
Male(no. = 1600)						
SBP(mmHg)	−0.903	0.133	<.001	−0.641	0.236	.007
DBP(mmHg)	−0.457	0.081	<.001	−0.312	0.146	.033
PP(mmHg)	−0.445	0.108	<.001	−0.329	0.187	.078
MAP(mmHg)	−0.606	0.088	<.001	−0.422	0.158	.008
Female(no. = 2139)						
SBP(mmHg)	−1.169	0.149	<.001	−0.780	0.219	.001
DBP(mmHg)	−0.598	0.085	<.001	−0.438	0.128	.001
PP(mmHg)	−0.571	0.125	<.001	−0.342	0.180	.058
MAP(mmHg)	−0.788	0.093	<.001	−0.552	0.140	<.001

Abbreviations: AMMW, appendicular muscle mass divided by body weight.; DBP, diastolic blood pressure; MAP, mean arterial pressure; PP, pulse pressure; SBP, systolic blood pressure; se, standard error;β, regression coefficient.

^a^ Model I: using simple linear regression model.

^b^ Model II: further adjusting for age, body waist, hypertension, anti‐hypertensive medicine, DM, smoking, and alcohol drinking.

## 4 DISCUSSION

The present study demonstrated that obese persons with higher AMMW are associated with lower BP, and the percentage of lean muscle mass (AMMW) is a good marker associated with BP (negative association) among the community‐dwelling elderly population in Taiwan.

To the best of our knowledge, this is the first study toward establishing the relationship between body composition, especially AMM to weight ratio, and BP in the community‐dwelling Taiwanese population. However, our study has a few limitations. First, the cross‐section study design limits us to identify the causal relationship between body composition and BP, though we assumed that physical training might affect body lean mass and BP. Second, we used estimated AMM by equation rather than instrumental measurement. Although the equation is evidence‐based to be superior to the BIA method,^18^ bias may exist as compared to instrumental measurement, but it should be limited. Third, since the history of chronic diseases, medications, and lifestyle patterns were collected by questionnaire under the assistance of a research technician, information bias still exists though it could be non‐differential. Finally, we could not obtain the information on the type of anti‐hypertension medications. There is a potential limitation of our study to further evaluate the effect of different anti‐hypertensive medicines on blood pressure and appendicular muscle mass ratio.

The AMMW was a good anthropometric marker that was negatively associated with BP and is a good potential marker in our study. However, several previous studies have demonstrated conflicting and inconsistent results. In Helsinki Birth Cohort Study, lean BMI (lean body mass divided by the square of height) had a positive association with BP.^17^ In the Tecumseh offspring study, Julius and associates reported a positive correlation between lean body mass with both SBP and DBP.^15^ The above studies use muscle mass and muscle mass index as variables which means the individual with overt muscle mass may influence the study results. The persons with overt muscle mass may come up with more total body mass and fat mass, which are known to be positively correlated to BP.^4^, ^17^

In this study, we used AMMW as a variable which showed a significant negative association to BP. In a large‐scale, longitudinal study conducted in Korea, Han and associates concluded that low skeletal muscle mass index (skeletal muscle mass (kg)/body weight (kg)X100)^20^ had an independent association with a greater risk of incident hypertension in men.^21^ A recent large‐scale study has shown an inverse association of BP to skeletal muscle mass percentage.^14^ Similar to the two studies, the current study used the ratio of lean body mass to total body mass as cofounding factor, showing a similar result. This could be explained that rather than muscle mass per se, the ratio of muscle mass to total body weight may be better associated with the risk of high BP and hypertension.

Central obesity was known to be related to incidence of hypertension^8^ and WC had direct association with BP.^7^ In our study, when adding AMMW into analysis, we found that persons with higher AMMW had lower SBP, DBP, and MAP in either central obese or non‐central obese persons. This result revealed that AMMW could be a better body composition indicator, which may help us to further stratify obese or central obese persons in the future studies.

In our study, the AMM is positively associated with BP, which means that AMM may be confounded with BW and BMI. As BMI, WHR, and AMM, may be confounded with each other in statistical models. We tried to evaluate the relationship between more detailed body composition and cardiometabolic risk factors, like AMMW and blood pressure in this study. By dividing AMM to body weight, the AMMW may dilute the influence of body weight, which also influenced BMI much more. The coefficient in the univariate regression of AMMW on BP did not show significant difference when compared to BMI. However, in the final multivariate model, AMMW revealed consistent inverse association and better than BMI even after adjusting for body waist. That is, using the AMMW ratio, there were consistent inverse results of percentage of body lean muscle mass and BP. It suggests that the percentage of lean muscle mass could be a more significant protective factor for hypertension (even after adjusting for WC). Previous studies had demonstrated that resistant training, which had the benefit of increasing muscle mass,^22^ could also reduce BP.^23^, ^24^ These results suggest that physical training toward increasing the lean mass ratio may be beneficial to BP and could help us to improve health promotion programs among the community‐dwelling elderly population. Also, further longitudinal study combined with interventional physical activities may be applied to provide more evidence to the current results.

## 5 CONCLUSION

In the current study, we concluded that obesity and central obesity were positively associated with BP. However, the AMMW was a protective factor to BP even in the central obesity persons. These findings help us to conduct health promotion programs among the elderly population in the future.

There is no source of funding (financial or material support) for our study and there is no funders took in the study. The authors received no specific funding for this work.

## CONFLICTS OF INTEREST

The authors have declared that there are no competing interests.

## Data Availability

Data will be available after contact with the corresponding author.
